# Intersectionality and Cognitive Impairment Risk in Older Persons With HIV: Age, Ethnicity, and LGBT Status

**DOI:** 10.1093/geroni/igaa057.2564

**Published:** 2020-12-16

**Authors:** Monica Rivera Mindt, Micah Savin, Angela Summers, Jordan Stiver, Alex Slaughter

**Affiliations:** Fordham University, New York, New York, United States

## Abstract

The Latinx population is disproportionately affected by HIV-infection and older Latinx persons living with HIV (PLWH) are at greater risk for neurocognitive impairment (NCI). However, no studies have examined whether intersectionality (including Lesbian Gay Bisexual Transgender [LGBT] status) increases NCI risk. This study investigated whether LGBT status increases NCI risk in 126 PLWH (Ages 19-73 years; 74% Male; 66% Latinx, 34% NHW) who completed a comprehensive NC battery. Domain average T-scores were based on demographically-corrected norms. Multiple regressions revealed that after accounting for covariates (cocaine use, premorbid IQ) and other dimensions of intersectionality (age, ethnicity), LGBT status significantly contributed to NCI risk in attention/working memory (B=-4.50, p=.01) and executive functioning (trend-level; B=-3.67, p=.06). LGBT status, a key dimension of intersectionality, should be considered in NC assessment of PLWH. Future research is needed to identify factors (e.g., discrimination) that may confer increased NCI risk in this population.

